# Supplementary material for: A Balanced Dietary Ratio of n-6:n-3 Polyunsaturated Fatty Acids Exerts an Effect on Total Fatty Acid Profile in RBCs and Inflammatory Markers in Subjects with Obesity
Source: Healthcare (Basel). 2023 Aug 18;11(16):2333. doi: 10.3390/healthcare11162333 (PMC10454033; doi:10.3390/healthcare11162333)
Supplement: Supplementary file 1 [file healthcare-11-02333-s001.zip › healthcare-2438574-supplementary.pdf]

Supplementary material

Variable	p-value	Rank	q-value	Discovery?
Weight (kg)	**0.008**	1	0.216	yes
Lean body mass (kg)	**0.012**	2	0.216	yes
BMI (kg/m^2)	**0.013**	3	0.216	yes
Water (kg)	**0.029**	4	0.300	yes
LDL-c (mg/dL)	**0.030**	5	0.300	yes
SM (kg)	0.052	6	0.406	no
BFM (kg)	0.062	7	0.406	no
Protein (%)	0.065	8	0.406	no
PUFA (%)	0.088	9	0.425	no
20:4 AA (g)	0.091	10	0.425	no
IL-8 (pg/mL)	0.097	11	0.425	no
AA (%)	0.102	12	0.425	no
Linoleic LA (g)	0.111	13	0.426	no
Linolenic ALA (g)	0.158	14	0.535	no
TG (mg/dL)	0.173	15	0.535	no
Insulin (μU/mL)	0.179	16	0.535	no
IL-6 (pg/mL)	0.182	17	0.535	no
DHA (g)	0.195	18	0.541	no
HOMA-IR	0.206	19	0.542	no
WC (cm)	0.229	20	0.557	no
DPA (%)	0.234	21	0.557	no
IL-13 (pg/mL)	0.268	22	0.571	no
VLDL-c (mg/dL)	0.275	23	0.571	no
EPA (g)	0.282	24	0.571	no
IL-12 (pg/mL)	0.286	25	0.571	no
Energy (kcal)	0.297	26	0.571	no
Fat (%)	0.343	27	0.634	no
Total n-6 (%)	0.361	28	0.634	no
Lipids (%)	0.368	29	0.634	no
Total cholesterol (mg/dL)	0.406	30	0.676	no
α linolenic acid (%)	0.437	31	0.693	no
Total sugar (g)	0.444	32	0.693	no
Cholesterol (mg)	0.466	33	0.706	no
Total n-3 (%)	0.569	34	0.836	no

22:5 DPA (g)	0.596	35	0.851	no
MUFA (%)	0.618	36	0.858	no
HDL-c (mg/dL)	0.728	37	0.910	no
EPA (%)	0.732	38	0.910	no
MIP1β (pg/mL)	0.757	39	0.910	no
MCP-1(pg/mL)	0.760	40	0.910	no
INFγ (pg/mL)	0.764	41	0.910	no
Adiponectin (ug/mL)	0.765	42	0.910	no
Carbohydrates (%)	0.803	43	0.933	no
SFA (%)	0.840	44	0.933	no
tFA (g)	0.840	45	0.933	no
Total PUFAs (%)	0.874	46	0.950	no
Glucose (mg/dL)	0.911	47	0.969	no
DHA (%)	0.956	48	0.981	no
Abdominal fat (kg)	0.977	49	0.981	no
Linoleic acid (%)	0.981	50	0.981	no
